# Plasticizers and Salt Concentrations Effects on Polymer Gel Electrolytes Based on Poly (Methyl Methacrylate) for Electrochemical Applications

**DOI:** 10.3390/gels8060363

**Published:** 2022-06-08

**Authors:** Carmen Rizzuto, Dale C. Teeters, Riccardo C. Barberi, Marco Castriota

**Affiliations:** 1 Department of Physics, University of Calabria Ponte Bucci, Cubo 33B, 87036 Rende, Italy; carmen.rizzuto@unical.it (C.R.); riccardo.barberi@fis.unical.it (R.C.B.); 2 CNR-Nanotec c/o Department of Physics, University of Calabria Ponte Bucci, 87036 Rende, Italy; 3 Department of Chemistry and Biochemistry, University of Tulsa, 600 S. College Ave., Tulsa, OK 74104, USA; dale-teeters@utulsa.edu

**Keywords:** gel, polymer electrolyte, impedance spectroscopy, ATR-FT-IR, DSC, linear sweep voltammetry, IR modes, plasticizer effect, monomer

## Abstract

This work describes the electrochemical properties of a type of PMMA-based gel polymer electrolytes (GPEs). The gel polymer electrolyte systems at a concentration of (20:80) % *w*/*w* were prepared from poly (methyl methacrylate), lithium perchlorate LiClO_4_ and single plasticizer propylene carbonate (PMMA-Li-PC) and a mixture of plasticizers made by propylene carbonate and ethylene carbonate in molar ratio 1:1, (PMMA-Li-PC-EC). Different salt concentrations (0.1 M, 0.5 M, 1 M, 2 M) were studied. The effect of different plasticizers (single and mixed) on the properties of gel polymer electrolytes were considered. The variation of conductivity versus salt concentration, thermal properties using DSC and TGA, anodic stability and FTIR spectroscopy were used in this study. The maximum ionic conductivity of σ = 0.031 S/cm were obtained for PMMA-Li-PC-EC with a salt concentration equal to 1 M. Ion-pairing phenomena and all ion associations were observed between lithium cations, plasticizers and host polymers through FTIR spectroscopy. The anodic stability of the PMMA-based gel polymer electrolytes was recorded up to 4 V. The glass temperatures of these electrolytes were estimated. We found they were dependent on the plasticization effect of plasticizers on the polymer chains and the increase of the salt concentration. Unexpectedly, it was determined that an unreacted PMMA monomer was present in the system, which appears to enhance ion conduction. The presence and possibly the addition of a monomer may be a technique for increasing ion conduction in other gel systems that warrants further study.

## 1. Introduction

Polymer electrolytes play an important role in the correct operation of electrochromic devices and lithium batteries. Their role is the ionic transport between two electrodes to balance the charges that arise during the redox process, which controls the electrochromic process. A good electrolyte system must exhibit high ionic conductivity, high electrochemical and thermal stability and high optical transmittance. Polar organic solvents make them of high dielectric constant and low viscosity, which promote ion migration through the electroactive material [1]. In the past, liquid electrolytes (LEs) were used as ion conductors inside electrochromic devices or lithium batteries [2]. In principle, they were made by dissolving a lithium salt in organic solvents such as propylene carbonate, ethylene carbonate, ethyl methyl carbonate, dimethyl carbonate and diethyl carbonate [3]. These solvents show low viscosity and low surface tension to ensure high ionic conductivities (10^−3^~10^−2^ S cm^−1^) [4,5]. Liquid electrolytes show some weaknesses that include low chemical stability, the possible formation of bubbles and the widespread use of large amounts of organic solvents that are necessary for their preparation [6].

Gel polymer electrolytes (GPEs) can circumvent the problems mentioned above. The gel electrolytes are made by a host polymer matrix (PMMA, PEO, PVC, PVDF, PVDF-HFP, PAN) that can host lithium salt (LiClO_4_, LiCF_3_SO_3_, LiBF_4_) dispersed into a solution of organic solvent (PC, EC, DEC, DMC) [1,7,8,9]. The diffusion of Li^+^ cations through the polymer matrix is obtained by using a high dielectric solvent that promotes the dissociation of the salt (Li^+^ and counter ions) in the polymeric matrix, which ensures the high ionic conductivity of the gel electrolyte [10]. Gel polymer electrolytes in different configurations have been proposed in electrochromic devices and lithium batteries: gel, blend and ionic liquid [11,12,13]. To improve the specific conductivity of the polymer electrolytes, the confinement of polymer electrolytes in the nanopores of alumina membranes has also been investigated [14].

Gel polymer electrolytes made by poly (methyl methacrylate) (PMMA) polymer are common and widely studied [15,16,17,18,19,20,21]. PMMA is commonly used for the fabrication of electrolytes because of its amorphous structure, which gives it many important properties like good solubility in the most organic solvents, flexible backbones, good ionic conductivity and high transparency, which is a crucial criterion for its application in electrochromic devices and lithium batteries [22].

In this work, PMMA was dissolved in two kinds of solutions: one made of lithium perchlorate and propylene carbonate (Li-PC). The second was made by dissolving the lithium salt in a mix of propylene carbonate and ethylene carbonate (Li-PC-EC). The PMMA additions to the solutions were conducted at room temperature, and transparent and homogeneous gels were obtained. Structural, electrochemical and thermal studies were conducted in order to characterize the gels obtained. The gel with the better performance would be the best candidate for electrochromic devices and lithium batteries.

## 2. Results and Discussion

### 2.1. AC Impedance and Ionic Conductivities

**Figure 1:**
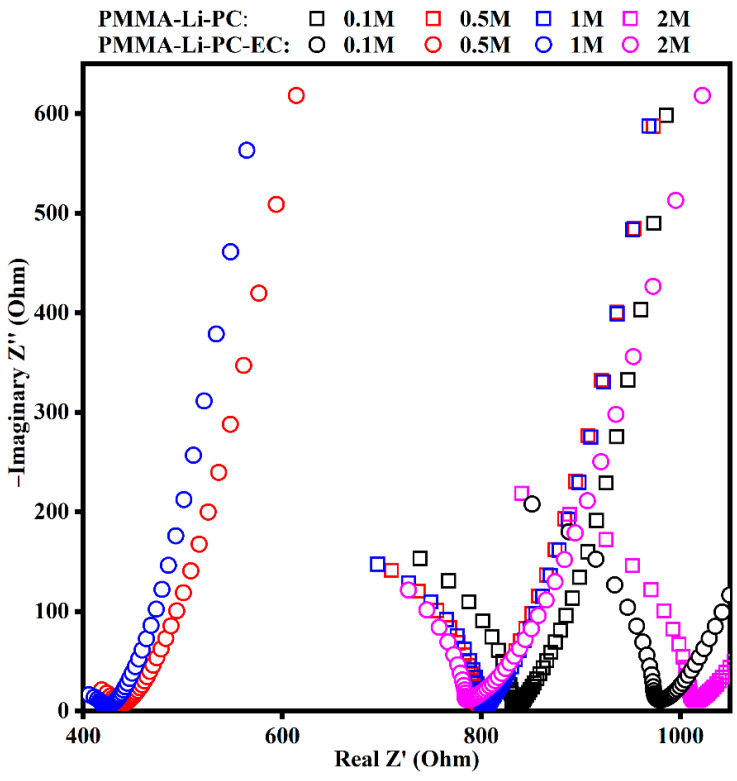
Nyquist plots in the range of 1 Hz to 1 MHz of gel polymer electrolytes PMMA-Li-PC (open squares) and of PMMA-Li-PC-EC (open circles) at different salt concentrations: 0.1 M (black), 0.5 M (red), 1 M (blue), 2 M (magenta).

The Nyquist plot of Figure 1 represents the data collected on the gel polymer electrolytes made by dissolving the PMMA in the solutions of lithium perchlorate and propylene carbonate (PMMA-Li-PC) and in the solutions of lithium perchlorate and propylene carbonate and ethylene carbonate (PMMA-Li-PC-EC).

The ideal semicircles that can be observed in Figure 1, at higher frequencies, represent the resistances of the studied systems. Ions are the only charge carriers and are thus responsible for the current in the polymeric system and are responsible for the total ion conductivity of the system [9]. The absence of the ideal semicircles in the Nyquist plot can be due to the polymer matrix’s presence with higher resistance with respect to the liquid solutions and the contribution of the electrode resistance [23]. The lower frequency region is under the diffusion control, which is the effect of the diffuse layer resistance of the dip cell.

**Figure 2:**
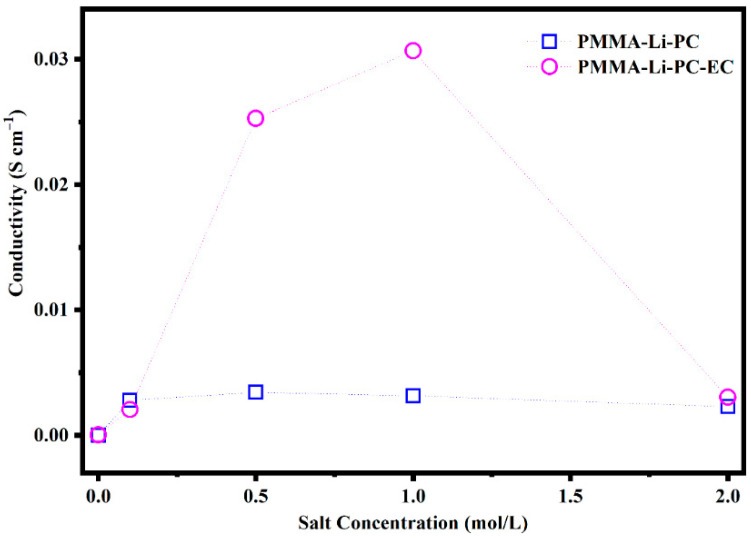
Trend of the ionic conductivities as a function of the salt concentration obtained on polymeric gel electrolyte.

The ionic conductivities of PMMA-Li-PC and PMMA-Li-PC-EC as a function of the salt concentration are shown in Figure 2.

As shown in Figure 2, the maximum value for the PMMA-Li-PC occurs at 0.5 M and is equal to 0.00343 S/cm. The maximum conductivity value is obtained for the systems PMMA-Li-PC-EC, when the salt concentration is equal to 1 mol/L, having a value of 0.031 S/cm. The PMMA-Li-PC value is comparable to values found for similar systems in the literature [16,17,18]. However, the value for the PMMA-Li-PC-EC is approximately an order of magnitude greater than other PMMA-Li-PC-EC systems studied [15,20,21]. A possible reason for this will be discussed later.

The results in Figure 2 are very interesting; as Mahbor saw in a similar case in previous research, the presence of the polymeric matrix and the use of the two solvents seem to promote the ionic conductivity in the gel. While Mahbor and coworkers saw a two-fold increase in conductivity for a 1 M salt solution, this work has almost a factor of 10 increase in conduction at the 1.0 m salt concentration. A possible reason for this will be discussed later.

Conductivity data as a function of lithium-ion concentration indicates that investigating interactions between electrolyte materials, host polymer and solvent plasticizer is very important to understanding the behavior of these systems. The following sections discuss these interactions.

### 2.2. FT-IR Interactions and Ion Associations

Infrared spectroscopy is a powerful instrument to confirm a polymer’s structure and investigate all possible interactions between electrolyte materials, host polymer, and plasticizer. All changes in vibrational modes are quantified in terms of shifting of band position, bandwidth, and intensity.

**Figure 3:**
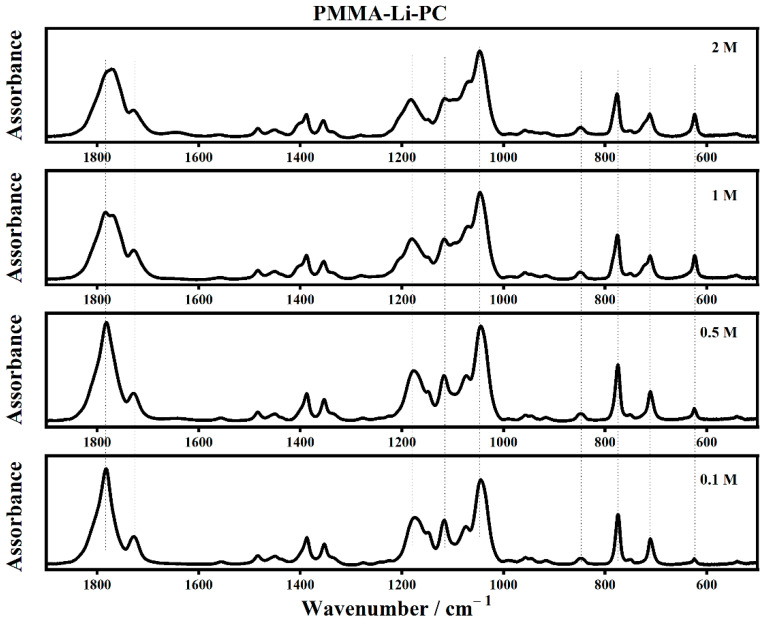
The IR spectra of PMMA-Li-PC for different salt concentrations in the frequency range between 500 cm^−1^ and 1900 cm^−1^.

**Figure 4:**
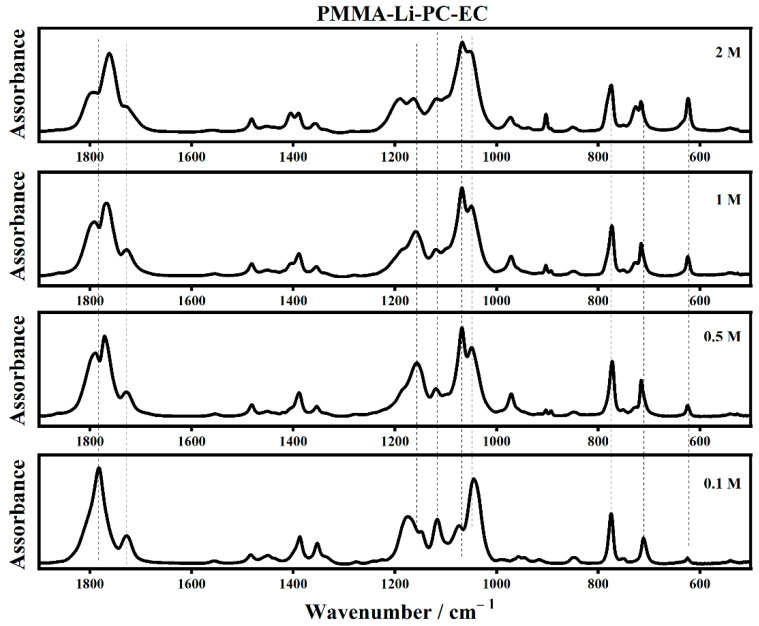
The IR spectra of PMMA-Li-PC-EC for different salt concentrations in the frequency range between 500 cm^−1^ and 1900 cm^−1^.

The infrared spectra of PMMA-Li-PC and PMMA-Li-PC-EC at different lithium perchlorate concentrations are shown in Figure 3 and Figure 4, respectively.

The assignments of some of the main IR modes collected on the polymeric systems are listed in Table 1.

The interactions between the lithium ions and the PC and EC molecules can be seen in the IR spectra because some of the relevant modes appear as bands upshifted in frequency, often called satellite bands [11,12].

**Figure 5:**
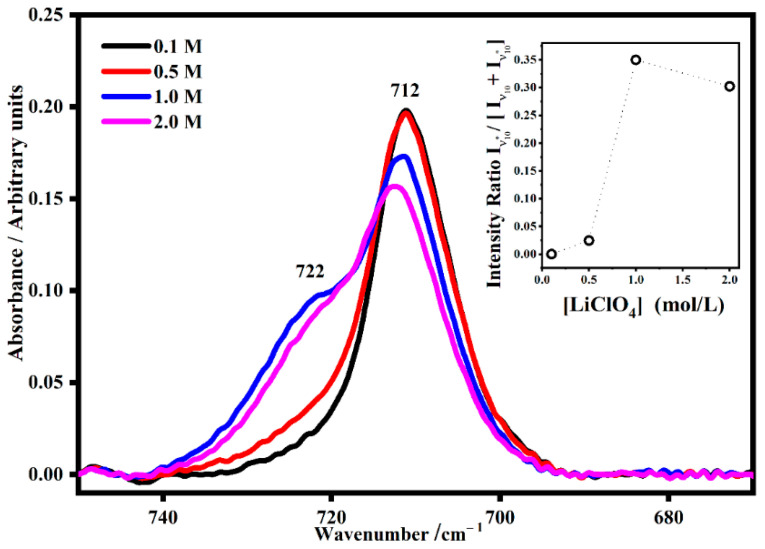
The IR mode denominated ν_10_ assigned to the symmetric ring deformation of propylene carbonate (712 cm^−1^) and its satellite band (ν_10_*) due to the symmetric ring deformation interaction with lithium ions (722 cm^−1^) in PMMA-Li-PC gel polymer electrolyte at different salt concentrations: 0.1 M, 0.5 M, 1 M and 2 M. In the inset, the trend of the intensity ratio I_ν10*_/[I_ν10_ + I_ν10*_] as a function of the salt concentrations.

To understand the relationship between the satellite bands and the salt concentration, the intensity ratios of the satellite band were normalized on the total intensity (sum of the intensity of the IR band and satellite intensity band). Propylene carbonate is a polar aprotic solvent with three different sites for electrostatic interaction with lithium cations; in particular, there are two ester-ether oxygen atoms and one carbonyl oxygen atom. In the polymeric gel PMMA-Li-PC, the band assigned to the symmetric ring deformation of propylene carbonate falls at 712 cm^−1^, and the relative satellite band at 722 cm^−1^ is due to the strong interaction between the PC ring and lithium ions (Figure 5) [24,25].

As shown in the inset plot in Figure 5, the trend of the intensity ratio I_ν10*_/[I_ν10_ + I_ν10*_] as a function of the concentration shows a maximum value for salt concentration equals to 1 M, which means that at this concentration the interactions between the PC ring and the lithium ions are maximum.

**Figure 6:**
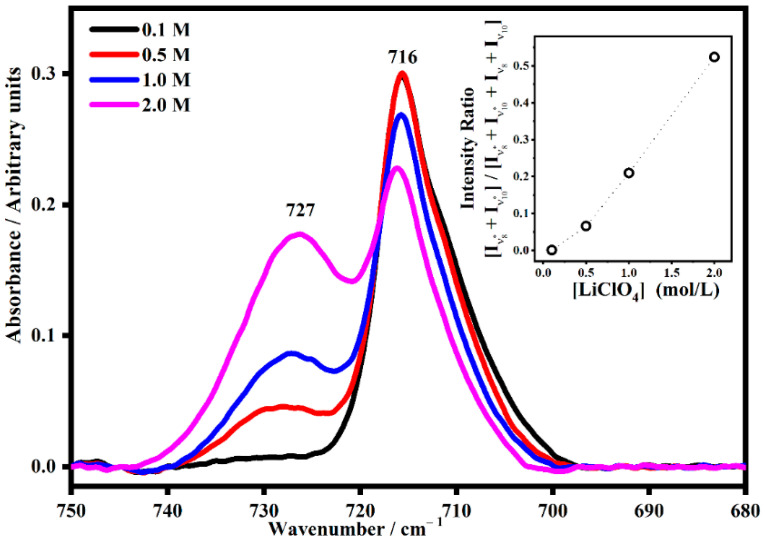
The IR band due to the sum of the PC mode denominated ν_10_ and the EC mode denominated ν_8_ (716 cm^−1^) and its satellite band (ν_8*_ + ν_10*_) due to the interactions between the plasticizers and the lithium ions (727 cm^−1^) in PMMA-Li-PC-EC gel polymer electrolyte at different salt concentrations: 0.1 M, 0.5 M, 1 M and 2 M. In the inset, the trend of the intensity ratio [I_ν8*_ + I_ν10*_]/[I_ν8_ + I_ν10_ + I_ν8*_ + I_ν10*_] as a function of the salt concentrations.

The effect of a mixture of propylene carbonate and ethylene carbonate (molar ratio 1:1) was also investigated by IR spectroscopy to understand the role of a combination of two plasticizers on the ion associations in PMMA-Li-PC-EC gel polymer electrolytes (Figure 6).

**Figure 7:**
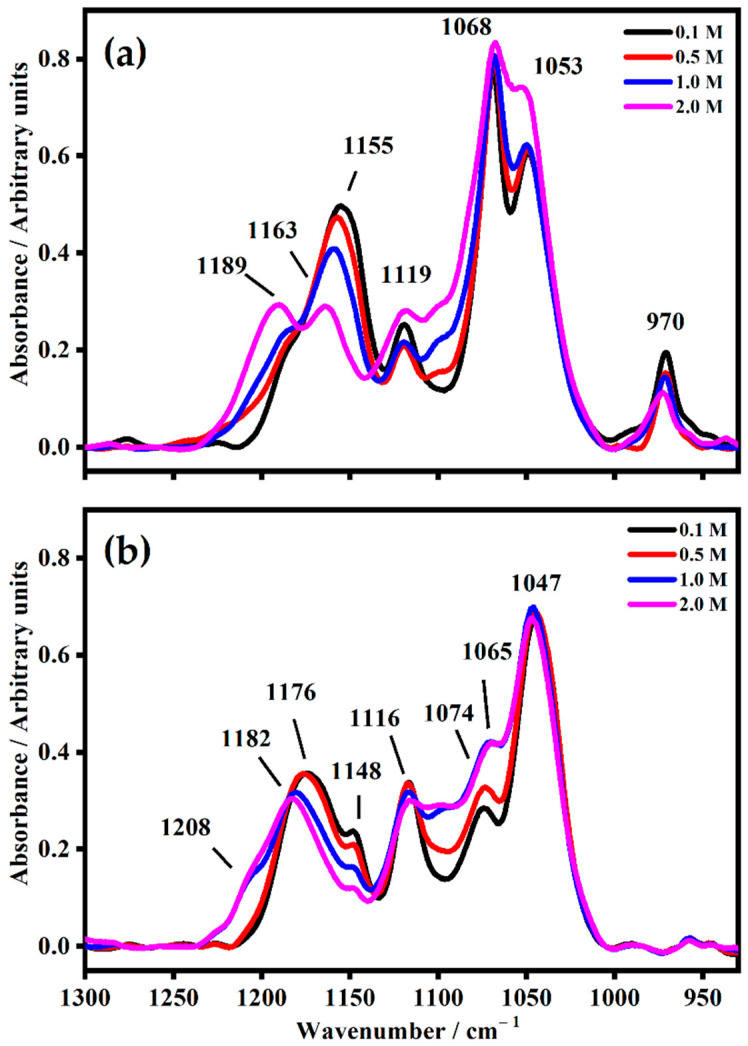
The IR spectra in the range between 930 cm^−1^ and 1300 cm^−1^ were collected on both polymeric gels with different salt concentrations 0.1 M, 0.5 M, 1 M and 2 M (**a**) PMMA-Li-PC-EC and (**b**) PMMA-Li-PC.

As shown in Figure 6, as the salt concentration increased, the satellite band at 727 cm^−1^ also increased. In particular, the inset figure shows the quantitative trend of the normalized [I_ν8*_ + I_ν10*_] directly increasing with the concentration. This can be interpreted as showing that the interactions between the plasticizers (EC and PC) molecules and the lithium ions increase as a function of the number of lithium ions present in the gel. Figure 7 shows the IR spectra collected on both the polymeric gels in the range between 930 cm^−1^ and 1300 cm^−1^.

In the 930 cm^−1^ to 1300 cm^−1^ region, as shown in Figure 7, it is possible to observe (on the bottom, for PMMA-Li-PC polymeric gel) that the O–C–O skeletal stretching IR band falls at 1176 cm^−1^ for 0.1 M and at 1182 cm^−1^ for 2 M (Figure 7) [26]. In addition, as the salt concentration increases, a new shoulder grew up at 1208 cm^−1^. The ring oxygen stretching is located at 1074 cm^−1^ at the lowest concentration (0.1 M), while at the highest concentration (2 M), this band downshifted at 1065 cm^−1^. It is due to the reduction of the bond’s polarity induced by the increased amount of lithium ions.

In the PMMA-Li-PC-EC gel polymer electrolyte (Figure 7b), the presence of the band at 970 cm^−1^ ascribed to the mode ν_4_ of the EC molecules is observed to decrease in intensity as the concentration of lithium is increased. This could be attributed to a general “tightening” of the matrix of the electrolyte with more interactions resulting from the additional lithium hindering symmetric skeletal stretching ν_4_ mode in the EC molecule. The most drastic decrease occurs at the 2 M concentration, where lithium interactions with the electrolyte matrix would have the potential to be the greatest. The variation of the number of lithium ions affected the IR frequency of the plasticizer bands, especially for the bands assigned to the stretching of C–O bond and wagging of C-H bond. In fact, the band at 1155 cm^−1^ for 0.1 M shifts at 1163 cm^−1^ for the solution 2 M where a new band at 1189 cm^−1^ occurs. The band at 1053 cm^−1^ assigned to the asymmetric O–C–O–O skeletal ring stretching of the plasticizer molecules increases drastically in frequency at the maximum concentration of 2 M.

**Figure 8:**
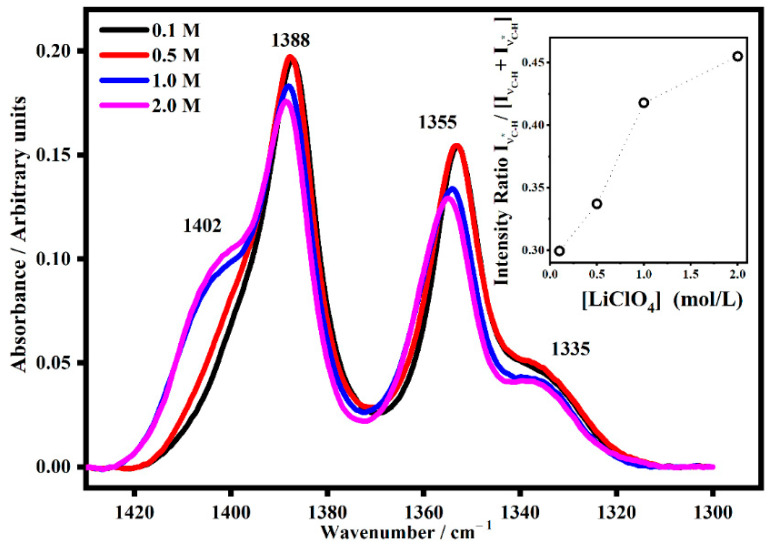
The IR band assigned to the CH_2_ wagging mode denominated ν_C-H_ (~1388 cm^−1^) and its satellite band at ν_C-H*_ (~1402 cm^−1^) in PMMA-Li-PC gel polymer electrolyte at different salt concentrations: 0.1 M, 0.5 M, 1 M and 2 M. In the inset, the trend of the intensity ratio I_νC-H*_/[I_νC-H_ + I_νC-H*_] as a function of the salt concentrations.

The spectra in Figure 8 shows the effect of the lithium salt concentration on the CH_2_ wagging deformation mode in the PMMA-Li-PC gel polymer electrolytes.

As can also be seen in Figure 8, the band at 1402 cm^−1^ occurs at higher concentrations. This band is due to the interaction between the CH_2_ groups and the lithium ions. In the inset the normalized trend of the intensity of the satellite band of CH_2_ wagging mode, I_νC-H*_/[I_νC-H_ + I_νC-H*_], is shown and it is possible to see the growing trend that indicates that the interactions increase as well as the concentration does.

**Figure 9:**
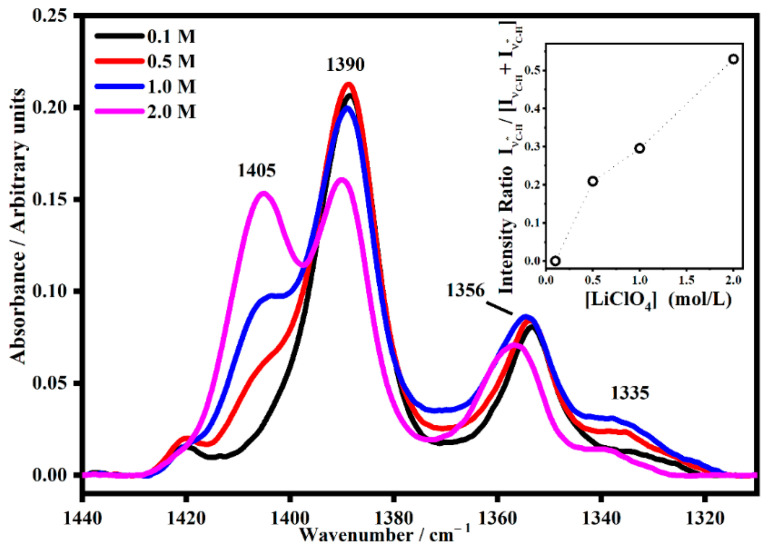
The IR band assigned to the CH_2_ wagging mode denominated ν_C-H_ (~1388 cm^−1^) and its satellite band at ν_C-H*_ (~1402 cm^−1^) in PMMA-Li-PC-EC gel polymer electrolyte at different salt concentrations: 0.1 M, 0.5 M, 1 M and 2 M. In the inset, the trend of the intensity ratio I_νC-H*_/[I_νC-H_ + I_νC-H*_] as a function of the salt concentrations.

Similar behavior was observed for the polymeric gel PMMA-Li-PC-EC, as can be seen in Figure 9.

As can be seen in Figure 9, the CH_2_ band falls at about 1390 cm^−1^ for the lower salt concentrations, but as the concentration increases, the interactions with lithium ions gradually become more evident with the growth of a new band (satellite) at 1405 cm^−1^. Such behavior is quantitatively shown in the inset of Figure 9, where the growing trend of the normalized intensity of the satellite band as a function of concentration is shown.

**Figure 10:**
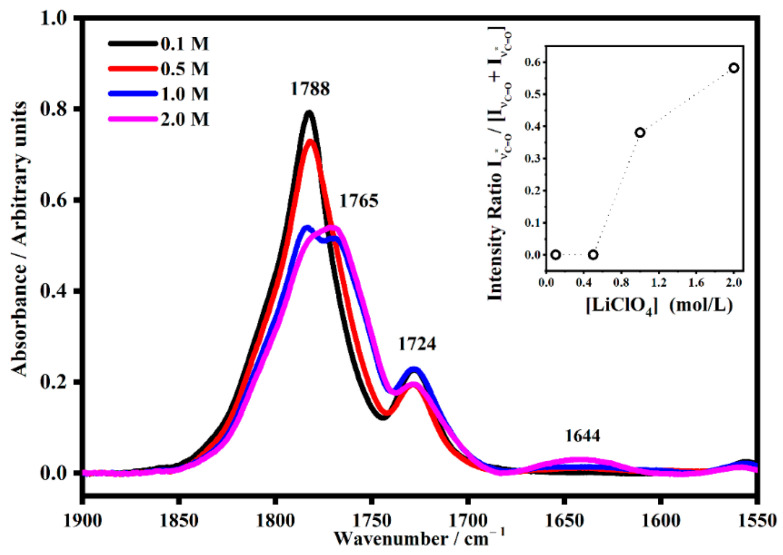
The IR band assigned to the C=O mode of free propylene carbonate denominated ν_C=O_ (~1788 cm^−1^) and its satellite band at ν_C=O*_ (~1765 cm^−1^) in PMMA-Li-PC gel polymer electrolyte at different salt concentrations: 0.1 M, 0.5 M, 1 M and 2 M. In the inset, the trend of the intensity ratio I_νC=O*_/[I_νC=O_ + I_νC=O*_] as a function of the salt concentrations.

At the higher frequency regions, it is possible to see the vibrational modes ascribed to the C=O bands. Figure 10 consists of the spectra collected on the PMMA-Li-PC polymeric gel electrolyte in the range between 1550 cm^−1^ and 1900 cm^−1^ at different salt concentrations: 0.1 M, 0.5 M, 1 M and 2 M.

The carbonyl stretching of “free” propylene carbonate falls at 1788 cm^−1^ (ν_C=O_); when it interacts with lithium ions it downshifts to 1765 cm^−1^ (ν_C=O*_) as shown in Figure 10 [24].

**Figure 11:**
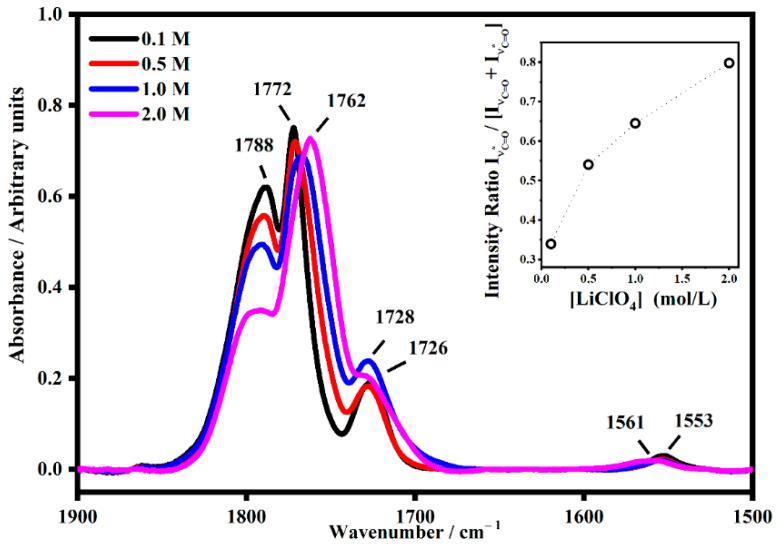
The IR band assigned to the C=O mode of free EC and PC carbonate denominated ν_C=O_ (~1788 cm^−1^) and its satellite band at ν_C=O*_ (~1772 cm^−1^ and ~1762 cm^−1^) in PMMA-Li-PC-EC gel polymer electrolyte at different salt concentrations: 0.1 M, 0.5 M, 1 M and 2 M. In the inset, the trend of the intensity ratio I_νC=O*_/[I_νC=O_ + I_νC=O*_] as a function of the salt concentrations.

As seen for the carbonyl group, the interactions with the lithium ions downshift the frequency of the relative satellite band. In addition, the PMMA has a carbonyl group that falls at about 1724 cm^−1^ and its interaction with lithium ions becomes visible for the samples at the highest salt concentration with the satellite band at 1644 cm^−1^. As shown in Figure 10, a remarkable effect is shown as a function of the concentrations in the band of the carbonyl of the PC molecules. In fact, also in the inset, it is possible to see that as the concentration along with the satellite bands increase due to the interaction between the PC molecules and the lithium ions increases. The same behavior was observed in the PMMA-Li-PC-EC polymeric gel, as shown in Figure 11. In this case, the bands of the free C=O of both plasticizers fell at 1788 cm^−1^ (ν_C=O_). This band shows the interaction with the lithium ions, which are dependent due to the concentration of the salt, as it downshifts in frequency where the lowest concentration the frequency is 1772 cm^−1,^ and for highest concentration the frequency is 1762 cm^−1^. This demonstrates that the plasticizer molecules interact with a higher number of lithium ions at higher concentrations. The modes that fall at about 1728 cm^−1^ were assigned to the polymeric structure and when interactions with the lithium ions occurred, a broad IR band at about 1553 cm^−1^ appeared.

**Figure 12:**
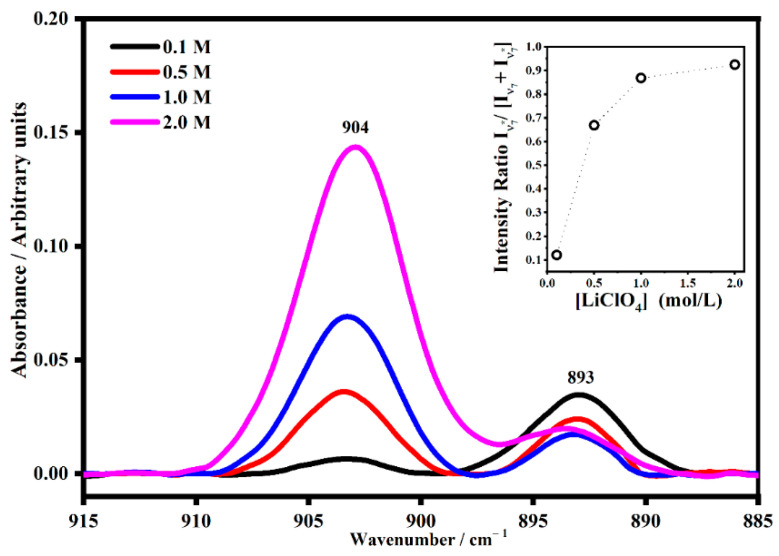
The IR band assigned to the skeletal ring breathing mode ν_7_ (~893 cm^−1^) of ethylene carbonate molecules and its satellite band at _ν7*_ (~904 cm^−1^) in PMMA-Li-PC-EC gel polymer electrolyte at different salt concentrations: 0.1 M, 0.5 M, 1 M and 2 M. In the inset, the trend of the intensity ratio I_ν7*_/[I_ν7_ + I_ν7*_] as a function of the salt concentrations.

Quite interesting is the band associated with the ν_7_ mode of the ethylene carbonate molecules that falls at 893 cm^−1^ (Figure 12). The satellite band at 904 cm^−1^ is due to the interaction between the EC molecules and the lithium ions. The quantitative estimation of the intensity ratio I_ν7*_/[I_ν7_ + I_ν7*_] as a function of the salt concentrations shows that at a low concentration, the satellite band is very small and becomes more intense as the concentration increases.

### 2.3. Ion Pairing

**Figure 13:**
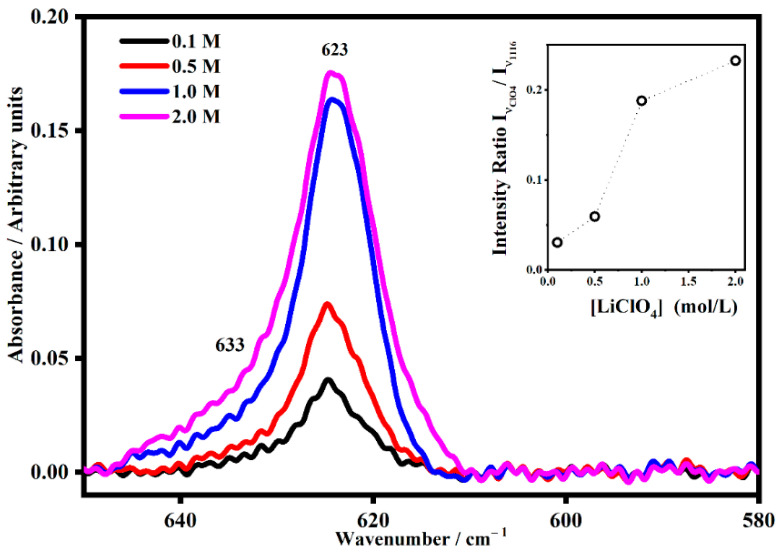
The IR band assigned to the ClO_4_^−^ anion I_νClO4_ (~623 cm^−1^) and its satellite band at I_νClO4*_ (~633 cm^−1^) in PMMA-Li-PC gel polymer electrolyte at different salt concentrations: 0.1 M, 0.5 M, 1 M and 2 M. In the inset, the Intensity Trend of the band at 623 cm^−1^ as a function of the salt concentrations for the PMMA-Li-PC polymeric blend system. The band at 623 cm^−1^, assigned to the free anion, was normalized by the intensity band at 1116 cm^−1^, which does not depend by the lithium perchlorate concentration.

**Figure 14:**
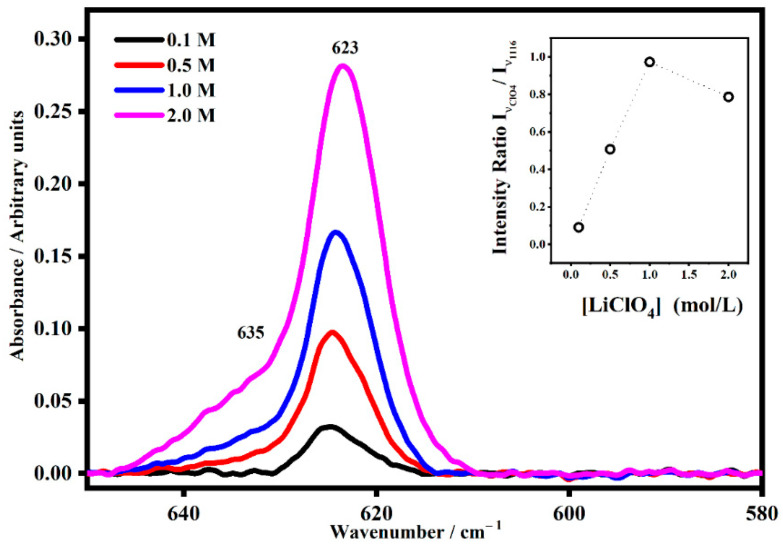
The IR band assigned to the ClO_4_^−^ anion I_νClO4_ (~623 cm^−1^) and its satellite band at I_νClO4*_ (~635 cm^−1^) in PMMA-Li-PC-EC gel polymer electrolyte at different salt concentrations: 0.1 M, 0.5 M, 1 M and 2 M. In the inset, the Intensity Trend of the band at 623 cm^−1^ as a function of the salt concentrations for the PMMA-Li-PC-EC polymeric blend system. The band at 624 cm^−1^, assigned to the free anion, was normalized by the intensity band at 1116 cm^−1^, which does not depend on the lithium perchlorate concentration.

Because of the presence of lithium ions, the four vibrational modes of ClO_4_^−^ could be perturbed and result in new satellite bands corresponding to a particular interaction. As is well known, the tetrahedral free anion perchlorate ClO_4_^−^ has nine vibrational modes, and under the tetrahedral symmetry (T_d_), four fundamental vibrational bands are allowed, where all of them are Raman active and only two are infrared active [24]. Changes in the band shape of these modes have been attributed to ion association [24]. The use of band fitting was useful to identify ion solvent interactions in the FT-IR spectra of the polymer electrolytes, and it led to the identification of four species: free ions ${\mathrm{Li}}^{+}{\mathrm{ClO}}_{4}^{-}$, solvent shared-separated ion pairs ${\mathrm{Li}}^{+}-\mathrm{solvent}-{\mathrm{ClO}}_{4}^{-}$, contact ion pairs and multiple ion aggregates ${\left\{{\mathrm{Li}}^{+}{\mathrm{ClO}}_{4}^{-}\right\}}_{n}$. The presence of each one is strong evidence of a Li^+^-plasticizer interaction. The free ClO_4_^−^ bands are located near 600 cm^−1^ and 1100 cm^−1^. For the PMMA-Li-PC-gelled systems, the band that describes the free ClO_4_^−^ is found at 623 cm^−1^ [27,28]. Starting with the 0.1 M system, with the increasing salt content, a new shoulder appeared located at 633 cm^−1^ for the PMMA-Li-PC system (Figure 13), and at 635 cm^−1^ for the PMMA-Li-PC-EC system (Figure 14).

The new bands at 633 cm^−1^ and 635 cm^−1^ are associated with ion pairs forming in the system as the salt concentration is increased [27,28]. The relative concentrations of the free ion, which are responsible for ion conduction, can be seen in the inset plots in Figure 13 and Figure 14. In these inset plots, the intensity of bands at 623 cm^−1^ assigned to the free anion was normalized to band intensities that do not depend on the lithium perchlorate concentration. The data shown in the inset plots is thus proportional to the concentration of the free ion in each system. In both the PMMA-Li-PC and PMMA-Li-PC-EC systems, the concentration of free ions is found to increase as the concentration of LiClO_4_ is increased. The change in free ion concentration can be compared to the ion conduction data shown in Figure 2. As might be expected, as the free ion concentration increases the ion conduction increase from 0.1 M to 0.5 M for all systems studied.

However, for all systems except the PMMA-Li-PC-EC system, the ion conduction starts decreasing at concentrations higher than 0.5 M. The PMMA-Li-PC-EC also exhibits a decrease in ion conduction but only at the highest concentration studied of 2 M. An increase in conduction as the concentration of free ion is increased is predicted since the dielectric theory of ionic conduction predicts that the conduction is proportional to the number of charge carriers and this is what is seen for lower ion concentrations as the concentration is increased. However, as the concentration is increased, the number of vacant coordinating sites in the electrolyte can be reduced, and this will start to reduce ionic conduction when a high enough concentration was reached [29]. This behavior was seen before for LiClO_4_ in a PEO polymer electrolyte system [30]. However, other factors such as chain dynamics can be responsible for this behavior. Understanding the greatly enhanced ion conduction for the PMMA-Li-PC-EC system compared to the PMMA-Li-PC system and the drastic drop of ionic conduction for the 2M PMMA-Li-PC-EC system (see Figure 2) in terms of segmental motion will be discussed later in this paper.

### 2.4. Linear Sweep Voltammetry

**Figure 15:**
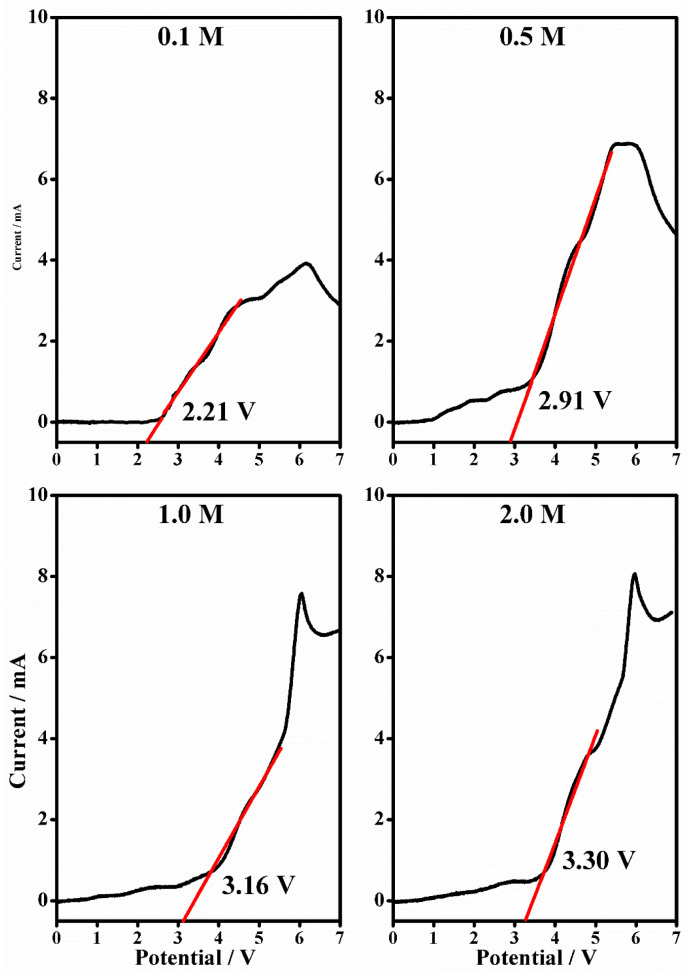
The Linear sweep voltammetry (black line) from 0 to 7 V and in the current range between 0 and 200 mA (scan rate 10 mV/s) and the anodic stability (relative linear fit, red line) of PMMA-Li-PC gel polymer electrolyte at different salt concentrations: 0.1 M, 0.5 M, 1 M and 2 M.

**Figure 16:**
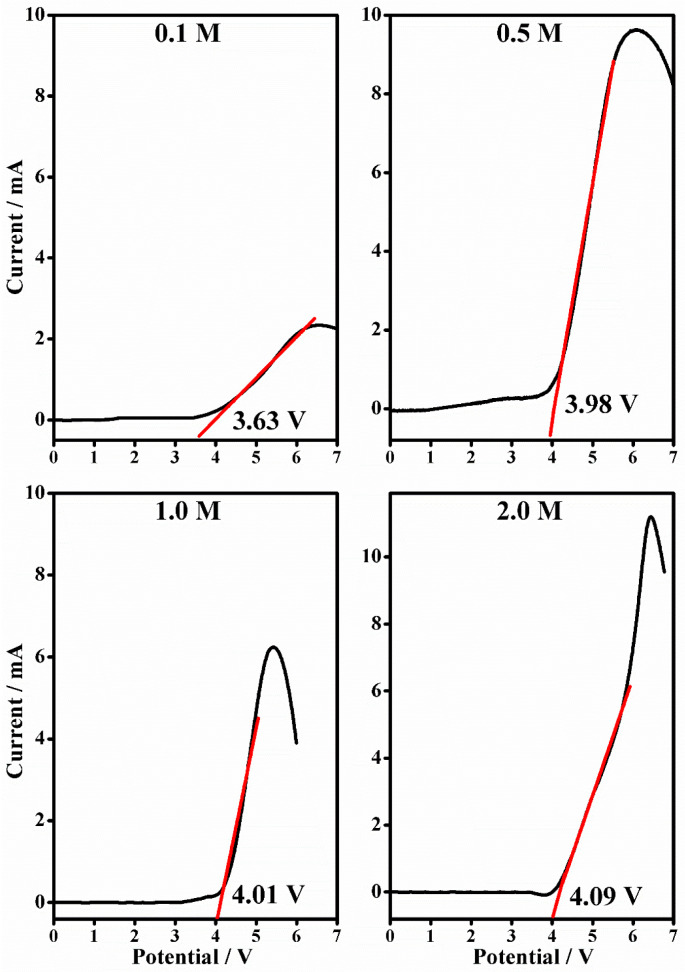
The Linear sweep voltammetry (black line) from 0 to 7 V and in the current range between 0 and 200 mA (scan rate 10 mV/s) and the anodic stability (relative linear fit, red line) of PMMA-Li-PC gel polymer electrolyte at different salt concentrations: 0.1 M, 0.5 M, 1 M and 2 M.

Linear sweep voltammetry was used to generate current voltage curves from 0 V to 7 V in order to define the anodic stability of the gel polymer electrolytes and to establish the electrochemical resistance of the material under increasing potential. The curves for PMMA-Li-PC (Figure 15) and PMMA-Li-PC-EC (Figure 16) at 0.1 M, 0.5 M, 1 M, 2 M salt concentrations were determined. As is typically done, one can assume that the anodic decomposition limit is the voltage where current flows through the cell. The anodic instabilities of polymeric gel electrolytes are evaluated by a sharp change in the current (slope) with respect to increased voltage scan in the anodic curves. The results are illustrated in Table 2.

The electrochemical investigations state that PMMA-based gel polymer electrolytes are stable up to 4 V, and their anodic stability increases with the increase of the LiClO_4_ content. Considering these values, one can assume the electrolyte is compatible with most high voltage electrodic couples of lithium composition. The limiting factor is ascribed to the decomposition of the electrolyte at the inert electrode. The value obtained is compatible with the results obtained elsewhere with similar systems [31].

### 2.5. Thermal Analysis

Differential Scanning Calorimetry (DSC) and Thermal Gravimetric Analysis (TGA) were performed on pure PMMA and polymeric gel electrolytes to investigate the thermal properties of the polymer and the effect that the plasticizers and different salt contents have on the thermal properties of PMMA-Li-PC and PMMA-Li-PC-EC systems at different concentrations: 0.1 M, 0.5 M, 1 M, 2 M.

**Figure 17:**
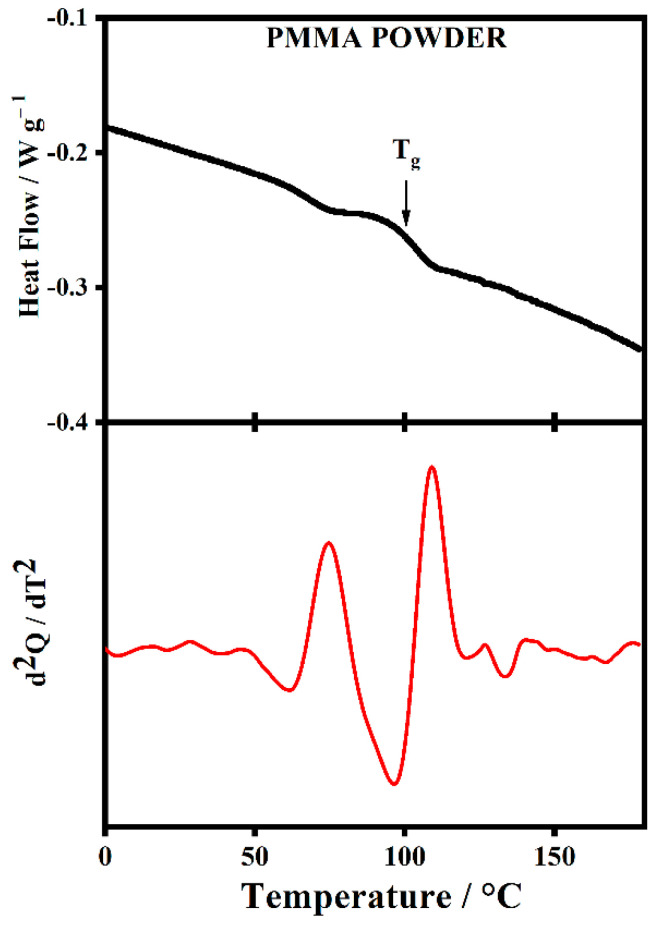
The Differential Scanning Calorimetry (DSC) curve (black solid curve) and the second derivative of the respective DSC signal (red dot curve) were collected during a heating process with a rate of 10 °C/min on PMMA pure powder.

Figure 17 shows the DSC thermogram of pure PMMA powder and the second derivative (assists in determining T_g_). It is possible to see the glass transition temperature of pure PMMA powder falls at 101.5 °C, which is at least 10 degrees lower than other values reported in the literature [32]. The significance of this will be discussed below.

**Figure 18:**
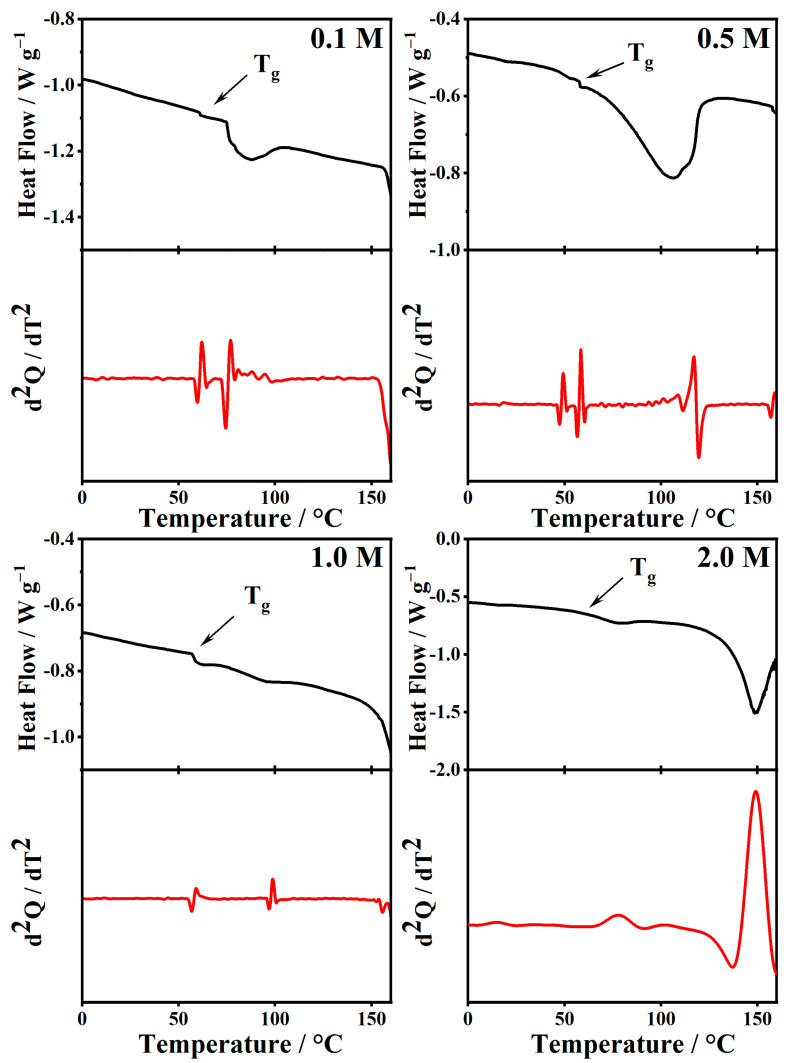
The Differential Scanning Calorimetry (DSC) curve (black solid curve) and the second derivative of the respective DSC signal (red dot curve) were collected during a heating process with a rate of 10 °C/min on PMMA-Li-PC gel polymer electrolyte at different salt concentrations: 0.1 M, 0.5 M, 1 M and 2 M.

**Figure 19:**
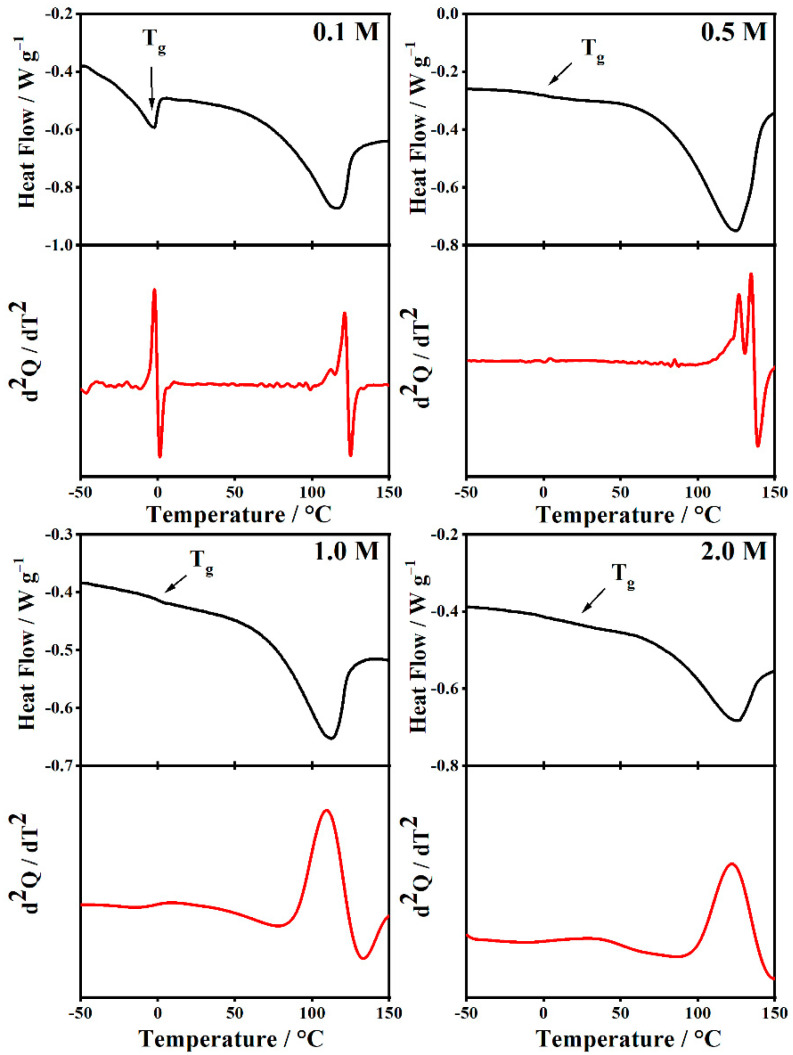
The Differential Scanning Calorimetry (DSC) curve (black solid curve) and the second derivative of the respective DSC signal (red dot curve) were collected during a heating process with a rate of 10 °C/min on PMMA-Li-PC-EC gel polymer electrolyte at different salt concentrations: 0.1 M, 0.5 M, 1 M and 2 M.

Figure 18 and Figure 19 show the DSC and their second derivate of the PMMA-Li-PC systems at the different concentrations of LiClO4: 0.1 M, 0.5 M, 1 M, and 2 M. In Figure 18 and Figure 19, endotherms around 100 °C can be seen in the thermograms. Bohnke and coworkers attributed endothermic peaks close to 100 °C to the vaporization of small amounts of water in the PMMA-Li-PC electrolyte systems they studied [17]. However, for the systems studied here, IR spectra, which are very sensitive to the presence of water, indicated that there was no water present in any of the systems. TGA data collected provides information as to what these endotherms are.

**Figure 20:**
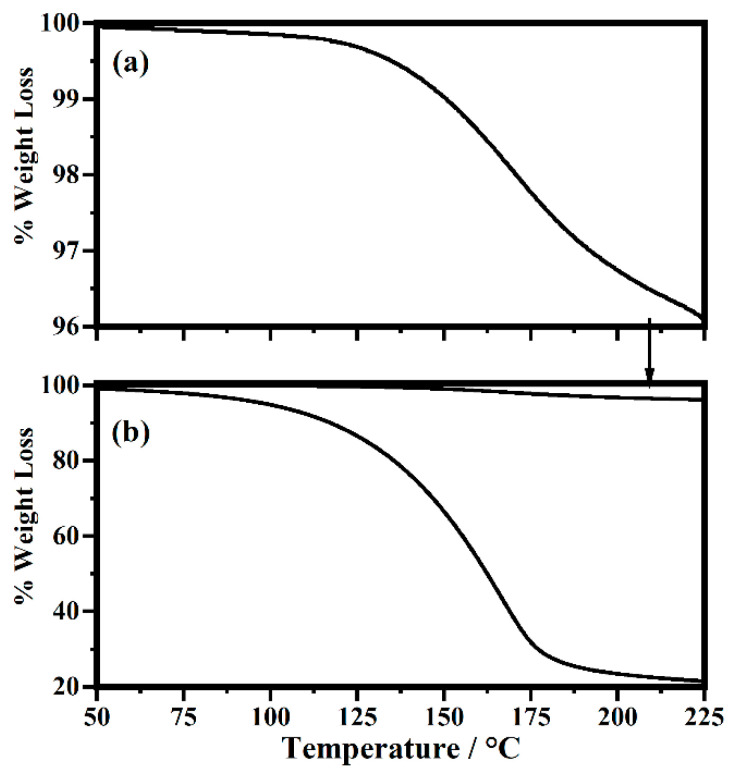
TGA data for PMMA polymer (**a**) and PMMA-Li (0.5 M)-PC-EC gel (**b**). The PMMA data have been plotted in the bottom image for comparison as indicated by the arrow.

Studies of pure PMMA polymer have found that it often contains unreacted methyl methacrylate (MMA) monomer, with a boiling temperature of 101 °C. Hirata and co-workers [33] concluded that volatilization of unreacted residual monomer can be responsible for weight loss in PMMA at temperatures below 210 °C. The amounts of MMA range from a fraction of 2% to over 5%, depending on the polymerization process forming the PMMA [34,35,36,37]. The observed weight loss seen in the upper image in Figure 20 appears to be due to the loss of unreacted monomer.

The lower image in Figure 20 is the TGA for a typical gel system studied in this work. A weight loss of approximately 70% is observed in the temperature range from 100 °C to 250 °C before further weight loss for other degradation processes occurs at higher temperatures (not shown). Thermal analysis studies [36,38] for PMMA polymer in this temperature range show weight loss can also be due to polymer chain degradation that results in monomer being formed. It is known that PMMA thermally degrades by three processes: a lower temperature process initiated by scissions of head-to-head linkages in the backbone forming monomer, a middle-temperature degradation of unsaturated end groups and the highest temperature degradation due to breaking of other backbone bonds. The head-to-head bond breakage occurs at the lowest temperature of the three degradations (above 100° but below 250 °C) because the bond dissociation energy of these linkages is estimated to be less than that of other C–C bonds in the backbone chain due to a large steric hindrance [36].

Comparing the TGA data for the PMMA and the gel, shown in Figure 20, one can see that while there is a large difference in the loss of weight between PMMA polymer and the gel, the temperature range for weight loss and shape of the curves are very similar indicating the weight loss process is associated with the same process: in this case, monomer volatilization. In the pure PMMA the size of the weight loss (~4%) indicates the loss is due to unreacted monomer being volatized [33]. In the gel, the large weight loss (70%) is due to head-to-head chain scission and volatilization of the MMA monomer formed by degradation. The PC plasticizers in these gels, PC and EC, appear to facilitate head-to-head linkage degradation and the resulting large weight loss.

Some work on gel electrolytes has attributed the weight loss in this range to the plasticizer PC being volatized [39]. This does not seem likely for the systems studied here since the boiling temperature for the two plasticizers used, PC (248 °C) and EC (243 °C), are much higher than where the loss starts at just above 100 °C. This loss would appear to be due again to loss of any unreacted MMA monomer present for PMMA and to scissions of head-to-head linkages in the PMMA backbone and then volatilization of the resulting monomer from the gels. All the endotherms in the DSC thermograms for the gels are located in this temperature range (see Figure 18 and Figure 19) and hence must be attributed to monomer volatilization. Importantly, the gels are stable up to temperatures around 100 °C, which means that they have a good use range for electrolyte applications.

Understanding the TGA data now allows focusing on information gained from glass transition data. The Tg have been labeled in Figure 18 and Figure 19. The T_g_ for the 0.1 M PMMA-Li-PC-EC has a slightly different form than other T_g_s seen in the thermograms but was confirmed by the cooling thermogram (not shown) to be the glass transition temperature. The experimental values of T_g_ from DSC curves for all systems are summarized in Table 3.

The addition of solvents such as propylene carbonate and ethylene carbonate has a plasticization effect on the polymeric chains and decreases the glass transition temperatures of the PMMA polymer by facilitating chain movement [19]. This is seen in this study since the glass transition temperatures for the PC, and PC-EC systems are lowered from 101.5 °C for pure PMMA.

For the PMMA-Li-PC system, the T_g_ drops around 40 °C with the addition of a PC plasticizer and the lithium salt. The T_g_ then remains relatively constant for the 0.1–1.0 M concentrations of LiClO_4_. While the PC lowers the glass transition temperatures in this concentration range, the fact that the T_g_ remains relatively constant indicates that there are few interactions between the polymer and lithium ions.

At the lowest concentrations, the lithium ions interact predominantly with the solvent PC molecules, and therefore the T_g_ reflects only interactions between the PMMA and the solvents molecules; the lithium ions are not significantly interacting with the polymeric chains [29]. However, when the concentration is increased to 2.0 M, the T_g_ increases, showing interactions now occurring between the lithium ions and polymer chains. For the polymeric gel at the highest salt concentration, the number of lithium ions becomes so high that ions can interact with solvent molecules, counter ions and polymeric chains [16]. The fact that the increasing salt concentration increases the interactions between the lithium ions, the solvent molecules, the counter ions and the polymeric chains was also shown in the IR investigations discussed above. Interactions with the polymeric chains induce an increase in the rigidity of the PMMA that is reflected in the increasing glass transition temperatures, T_g_, as shown in Table 3. This increase indicates a decrease in polymer chain segmental motion. Since the Vogel–Tammann–Fulcher (VTF) model for ion transport in polymer electrolytes states that ion transport is assisted by polymer segmental motion, a higher T_g_ associated with less segmental motion [40] could be partially responsible for the small reduction in conductivity for the 2M PMMA-Li-PC system seen in Figure 2. This factor and the small decrease in free ion concentration shown by IR data (see Figure 13 inset plot) probably both contribute to this small reduction in ionic conductivity.

The combined solvent PMMA-Li-PC-EC has the most reduction in glass transition temperatures, which in general, by the VTF model, would favor higher ionic conduction. While the reduction in the glass temperature is much greater than in the PMMA-Li-PC system, the T_g_s also remain relatively constant for 0.1–1.0 M concentrations of LiClO_4_ ranging from −1.94 to 7.80 °C. This again indicates that there are little interactions between the polymer chains and lithium ions for these concentrations. When comparing these data to the ionic conduction data shown in Figure 2, the increase in ionic conductivity compared to the PMMA-Li-PC could be facilitated by the much lower T_g_ values for the PMMA-Li-PC-EC gel. However, since T_g_ is a relative constant up to the 1.0 M concentration, the change in ionic conduction appears to be due to the increase in free ion concentration with the increase in LiClO_4_ concentration, as shown in the inset plot in Figure 14 and not a change in segmental motion. When the concentration is increased to 2.0 M, the free ion concentration continues to increase (Figure 14 inset plot); however, the concentration of salt is great enough that it can now interact with the polymer chains, raising the T_g_ by almost 30 °C to approximately 36 °C thus hindering segmental motion. The IR data discussed above and shown in Figure 10 also confirms that the lithium ions have strong interactions with the PC chains at this higher concentration. It is this reduction in segmental motion and not the concentration of free ions that greatly lowers the ionic conduction for the 2 M system, as seen in Figure 2.

Results from the thermal analysis can help to explain the relatively higher value of ion conduction for the PMMA-Li-PC-EC system compared to other studies done in the literature. As discussed above, the PMMA used in this study could have as much as 4% unreacted monomer in the PMMA polymer. The very nonpolar monomer PMMA remaining in the PMMA polymer could have two effects. First, it is known that MMA monomer in PMMA acts as a plasticizer [34,41,42]. In addition to the PC-EC plasticizers, the presence of the monomer plasticizer would further lower the Tg of the PMMA, increasing ion conduction. The presence of the unreacted monomer acting as an additional plasticizer could be the reason that the Tg for the pure PMMA used in our work was found to be lower than other Tg values for PMMA found in the literature (see above). Interestingly, studies of PMMA-LiClO_4_-PC and PMMA- LiClO_4_-PC-EC systems described in the literature [15,16,17,18,19,20,21] have all heated the PMMA polymer used to prepare gels to temperatures of 55 to 100 °C under vacuum for up to 48 h. This heating under vacuum was done to remove any water in the PMMA used. Since our IR data indicated that the PMMA used here did not contain water, the PMMA used in this study was not heated. It is well known in the literature [35,37,43,44,45], and our TGA data presented in this work has shown that heating PMMA polymer will remove unreacted monomer. This is why DSC endotherms associated with the vaporization of MMA (see above) and TGA weight loss data, Figure 20, show the presence of monomers in our systems. This is compared to other studies in the literature on these systems using thermal analysis techniques [3,4,5] that do not show DSC endotherms at this temperature, i.e., there was no monomer in their samples because of heating under vacuum. Again, the presence of the monomer in the samples could further lower the Tg, increasing ion conduction. Second, the MMA molecule is very nonpolar. This nonpolar molecule acting as a plasticizer and being in close proximity to the PMMA backbone would further hinder interactions between the polymer chains and lithium ions freeing ions to move in the plasticizer molecules in the gel. As described above, only when the concentration is at 2.0 M is the concentration of lithium ions high enough so that interactions with the polymer chains are forced, raising the T_g_ and thus hindering segmental motion, lowing ion conduction. The concept of polymer monomer acting as a plasticizer is interesting and warrants further investigation.

Table 4 shows a summary of the glass transition temperatures (Tg), Ionic Conductivity and Anodic Stability of the PMMA-Li-PC and PMMA-Li-PC-EC polymeric gel electrolytes at different salt concentrations: 0.1 M, 0.5 M, 1 M and 2 M. As described above, the trend of the Tg shown in Table 4 indicate that not all the MMA monomer is polymerized but that some works as plasticizer promoting the ion conduction in PMMA-Li_PC-EC polymer electrolyte, which results are the highest for the 1 M gel. The working stability of polymeric gel electrolyte remains for applied voltage up to 4 V.

## 3. Conclusions

In this work, PMMA-Li-PC and PMMA-Li-PC-EC gels were studied at LiClO_4_ concentrations of 0.1 M, 0.5 M, 1 M and 2 M.

The highest conductivity value of σ = 0.031 S cm^−1^ was obtained in the PMMA-Li-PC-EC 1M system.

Linear sweep voltammetry shows the anodic stability of the gels, which is about 4 V in the investigated potential range.

A detailed IR spectroscopic study of PMMA-Li-PC and PMMA-Li-PC-EC systems showed that increasing the salt concentration increases the interactions between the lithium ions, the solvent molecules, the counter ions, and the polymeric chains. IR data support the results obtained by thermal analysis, which indicates that the systems made with ethylene carbonate, PMMA-Li-PC-EC compared to the gel with only PC have a lower T_g_ promoting segmental motion and have good concentrations of free ion. Both segmental motion and free ion concentrations were found to be important in promoting high ionic conduction of 0.031 S·cm^−1^. An interesting result of thermal analysis studies was that residual MMA monomer was present in the PMMA used to make the gels. Since the monomer is known to plasticize PMMA, the resulting increase in polymer chain segmental motion could be responsible for the very high ion conduction for the PMMA-Li-PC-EC system seen here when compared to other work done in the literature.

As a result of the present study, future research will be focused on PMMA-Li-PC-EC polymeric gel electrolytes in the manufacturing of electrochromic devices for energy-saving applications.

## 4. Materials and Methods

### 4.1. Materials

Poly (methyl methacrylate) (PMMA) (Mw = 120,000) was purchased from the Sigma-Aldrich Company (St. Louis, MO, USA). Lithium perchlorate (LiClO_4_), propylene carbonate (PC) and ethylene carbonate (EC) were obtained from the Sigma-Aldrich Company. All the compounds were preserved inside a glove box to prevent absorbing moisture prior to preparation and to further characterizations.

### 4.2. Preparation of Liquid and Gel Polymer Electrolytes

All the samples were prepared in an argon atmosphere in a glove box (MBRAUN-Unilab, Garching, Germany). Lithium salt was used to make two kinds of solutions: one made by the lithium perchlorate dissolved in propylene carbonate (Li-PC) and the second made by dissolving the salt in a mix of propylene carbonate and ethylene carbonate (Li-PC-EC), with a molar ratio of the two solvents 1:1. Solutions with different salt concentrations were made: 0.1 M, 0.5 M, 1 M and 2 M, for both of the systems. These concentrations indicate that the mechanism of the conduction remains the same for all the concentrations used; in the case of higher concentrations, the possibility of having clusters increases. For both of the studied gels, the conduction decreased at 2 M; hence, higher concentrations would be expected to have even lower conductivities and are not of much interest. The gel polymer electrolytes were obtained by mixing 20% *w*/*w* of PMMA (Mw = 120,000) and 80% *w*/*w* of each solution: PMMA-Li-PC and PMMA-Li-PC-EC. The percentages ratios, with 20% of PMMA, were found by Bohnke and coworkers [16] to have a more liquid-like behavior and hence a higher conductivity than concentrations of PMMA with higher percentages. Starting with this higher conducting polymer base for the gels provided the best starting point for modification to make the best electrolytes in electrochromic devices. Finally, each mixture was stirred and heated at 100 °C for one hour. At the end of this process, each solution was left to rest for one night.

### 4.3. Characterization of Gel Polymer Electrolytes

AC impedance data of each sample were collected inside the glovebox by using a commercial dip cell, mercury-free Conductivity Probes (Fisherbrand accumet, 300 Industry Drive, Pittsburgh, PA, USA) with a Nominal Cell Constant of 1.0 cm^−1^. The AC impedance data were made using a Solartron SI 1260 Impedance/Gain Phase Analyser (Solartron Analytical, Hampshire, UK) with a 1296 interface. SMaRT Impedance Measurement Software (Solartron Analytical, Farnborough, UK) was used to scan from 1 Hz to 1 MHz, while the AC voltage was set at 0.1 V. ZView software 3.4 was used to analyse the ac-Impedance data. An equivalent circuit was used to model ionic conduction in the system and determine ionic conductivity resistance values. The circuit is composed of different electric elements: a resistor R1 which describes the electrode resistance, a capacitor C and a second resistor R2 in parallel, which are attributed to the electrolyte resistance and a Warburg impedance W1 in series to R2, which is related to the diffuse layer resistance. Resistivity and Ionic conductivity values were calculated using the following equations, ρ = R/Nominal cell constant (Ω·S) and σ = 1/R (S·cm^−1^) where R is the bulk resistance of the electrolyte, estimated by the fitting model of the Nyquist plot of complex impedance for liquid and gel polymer electrolytes. The impedance of each sample was measured three times to ensure data reproducibility.

The ATR-FTIR spectra were collected by using Thermo Scientific Nicolet (Waltham, MA, USA) iS50 Fourier Transform Infrared spectrometer using an attenuated total reflection attachment in the absorbance mode in the range between 400 cm^−1^ and 4000 cm^−1^. The ATR cell had a ZnSe crystal cut at 45°.

The average depth of penetration of the infrared radiation, dp, was calculated by using:

(1) $$\;d_{p}=\frac{\lambda }{\;2\pi n_{1}{\left(sin^{2}\theta -n_{21}^{2}\right)}^{2}}\;$$

where *λ* is the wavelength of the radiation in the air, *θ* is the angle of incidence (45°), *n*_1_ is the refractive index of the ATR crystal (2.4) and *n*_21_ is the ratio of the refractive index of the sample to that of the ATR crystal (0.605). Using these values, an average depth of infrared radiation in the sample was determined to be approximately 1.0 mm in the region of interest. The spectral resolution of the data is 1 cm^−1^. All the measurements were made at room temperature (25 °C).

The DSC of the gel electrolyte samples was carried out on DSC2500 (TA instruments, New Castle, Germany) in the range between −90 °C and 180 °C with a heating cooling rate of 10 °C/min under a constant nitrogen flow (50 mL/min) to avoid any contact of atmospheric moisture. Each sample was put inside an aluminum sample pan. The following DSC setup was used: (1) equilibrate to 35 °C, (2) cooling to −90 °C with cooling rate of 10 °C min^−1^, (3) heating to 180 °C with heating rate of 10 °C/min, (4) and equilibrate at 35 °C with cooling rate of 10 °C min^−1^. The glass transition temperature, T_g_, was obtained by the discontinuity in the second derivative of the heat flow from DSC thermograms. TGA data were collected on a TA Instruments’ TGA 5500 under nitrogen at 2.5 °C/min.

The anodic stability of each sample was determined from linear sweep voltammetry. The voltammetry measurements were carried out on gel polymer electrolytes in the current range from 0 and 200 mA at a scan rate of 10 mV/s with a Potentiostat PARSTAT 4000+ (AMETEK Scientific Instruments, Oak Ridge, TN, USA), and using an electrochemical cell composed of two steel plates (7.6 cm × 1.27 cm × 0.05 cm).

## Figures and Tables

**Table 1:** Assignments of the main IR modes of the spectra collected on the polymeric gel denominated: PMMA-Li-PC and PMMA-Li-PC-EC.

ETHYLENE CARBONATE		
Wavenumber/cm^−1^	Assignments	Mode
714	Ring bending	ν_8_
770	Rocking in-phase of CH_2_	ν_22_
892	Skeletal ring breathing	ν_7_
970	Symmetric skeletal stretching	ν_4_
1007	Rocking out of phase of CH_2_	ν_11_
1060	Symmetric skeletal stretching (ring breathing)	ν_5_
1150	Stretching of (C–O) + Wagging of C–H	ν_10_
1217	Twisting out-of-phase of CH_2_	ν_21_
1231	Twisting in-phase of CH_2_	ν_19_ + ν_8_
1391	Wagging out-of-phase of CH_2_	ν_4_
1420	Wagging in-phase of CH_2_	ν_15_
1486	Bending of CH_2_	ν_2,_ ν_14_
1553	Overtone of 770 cm^−1^ band	2 ν_22_
1682	Overtone of 970 cm^−1^ and 714 cm^−1^ bands	ν_4_ + ν_8_
1789	Stretching of C=O	2 ν_7_
**PROPYLENE CARBONATE**		
711	Symmetric ring deformation	ν_10_
774	Ring deformation	ν_18_
848	Stretching of CH_3_ + Ring (Ring breathing)	ν_9_
917	Rocking of CH_3_	ν_32_
948	Rocking of CH_3_	ν_31_
957	Ring Stretching + Bending of CH_3_	ν_8_
1043	Asym. Ring Stretch. + Bend. of C–H + Twist. of C–C	ν_6_
1075	Asymmetric Ring Stretching	ν_7_
1116	Wagging of C–H + Bending of C–H	ν_20_
1172	Stretching of O–C + Wagging of C–H	ν_5_
1353	Symmetric bending of C–H	ν_16_
1387	Wagging of C–H + Bending of C–H	ν_30_
1450	Bending of C–C-H in CH_3_	ν_29_
1484	Umbrella of C–H in CH_3_	ν_15_
1779	Stretching of C=O	ν_1_
	**PERCHLORATE**	
625	Free ClO_4_^−^	ν_4_
944	ClO_4_	ν_1_
1089	Pure LiClO_4_	ν_3_
	**PMMA**	
750	Asymmetric rocking of CH_2_ (skeletal mode)	–
840	Symmetric rocking of CH_2_	–
968	Rocking of (α-CH_3_)	–
987	Rocking of (O–CH_3_)	–
1063	Stretching of C–C (skeletal mode))	–
(1142–1191)	Stretching of C–O–C	–
(1239–1266)	Stretching of C–O bond or the C–C–O (ν_4_)	–
1387	Symmetric bending of C–H in (α-CH_3_)	–
1433	Symmetric bending of (C–H) of O–CH_3_	–
1447–	Asymmetric bending of (C–H) in (α-CH_3_)	–
1479	Scissoring of CH_2_	–
1721	Symmetric stretching of C=O	–

**Table 2:** Anodic stability from 0 to 7 V and in the current range between 0 and 200 mA (scan rate 10 mV/s) of polymeric gel electrolyte at different salt concentrations: 0.1 M, 0.5 M, 1 M and 2 M.

[LiClO_4_] (mol/L)	Anodic Stability (V)	
	PMMA-Li-PC	PMMA-Li-PC-EC
**0.1**	2.21	3.36
**0.5**	2.91	3.98
**1.0**	3.16	4.01
**2.0**	3.30	4.09

**Table 3:** Glass transition temperatures were obtained from the DSC studies performed on PMMA and PMMA-Li-PC, and PMMA-Li-PC-EC polymeric gel electrolytes at different salt concentrations: 0.1 M, 0.5 M, 1 M and 2 M.

[LiClO_4_] (mol/L)	PMMA	PMMA-Li-PC	PMMA-Li-PC-EC
	T_g_ (°C)	T_g_ (°C)	T_g_ (°C)
**0.0**	101.5	-	-
**0.1**	-	61.9	−1.94
**0.5**	-	58.3	4.41
**1.0**	-	59.0	7.80
**2.0**	-	78.1	36.3

**Table 4:** Summary of the main results shown above for PMMA-Li-PC and PMMA-Li-PC-EC polymeric gel electrolytes at different salt concentrations: 0.1 M, 0.5 M, 1 M and 2 M: glass transition temperatures (T_g_), Ionic Conductivity and Anodic Stability.

[LiClO_4_] (mol/L)	PMMA-Li-PC			PMMA-Li-PC-EC		
	T_g_ (°C)	Ionic Conductivity (S/cm)	Anodic Stability (V)	T_g_ (°C)	Ionic Conductivity (S/cm)	Anodic Stability (V)
**0.1**	61.9	0.00278	2.1	−1.94	0.00206	3.63
**0.5**	58.3	0.00343	2.91	4.41	0.02527	3.98
**1.0**	59.0	0.00314	3.16	7.80	0.03068	4.01
**2.0**	78.1	0.00229	3.30	36.3	0.00304	4.09

## Data Availability

The data presented in this study are available on request from the corresponding author.
